# Efficacy of endoscopic surveillance in the detection of local recurrence after radical rectal cancer surgery is limited? A retrospective study

**DOI:** 10.1186/s12957-021-02413-0

**Published:** 2021-10-21

**Authors:** Michal Jankowski, Wojciech M. Wysocki, Manuela Las-Jankowska, Karol Tkaczyński, Dorian Wiśniewski, Dariusz Bała, Wojciech Zegarski

**Affiliations:** 1 Chair of Surgical Oncology, Ludwik Rydygier’s Collegium Medicum in Bydgoszcz, Nicolaus Copernicus University, Toruń, Poland; 2 Department of Surgical Oncology, Oncology Center—Prof Franciszek Łukaszczyk Memorial Hospital, Romanowskiej 2 Street, 85-796 Bydgoszcz, Poland; 3 Department of Surgery, Faculty of Medicine and Health Sciences, Andrzej Frycz Modrzewski Krakow University, Gustawa Herlinga-Grudzińskiego 1 Street, 30-705 Kraków, Poland; 4 Department of General, Oncological and Vascular Surgery, 5th Military Clinical Hospital in Kraków, Wrocławska 1-3 Street, 30-901 Kraków, Poland; 5 National Institute of Oncology, Maria Skłodowska-Curie Memorial, Scientific Editorial Office, W.K. Roentgena 5 Street, 02-781 Warszawa, Poland; 6 Department of Clinical Oncology, Oncology Center—Prof Franciszek Łukaszczyk Memorial Hospital, Romanowskiej 2 Street, 85-796 Bydgoszcz, Poland

**Keywords:** Surveillance after radical surgery, Rectal cancer, Local recurrence, Endoscopy, Computed tomography, Magnetic resonance imaging

## Abstract

**Background:**

Rectal cancer, one of most common neoplasms, is characterized by an overall survival rate exceeding 60%. Nonetheless, local recurrence (LR) following surgery for rectal cancer remains a formidable clinical problem. The aim of this study was to assess the value of postoperative endoscopic surveillance (PES) for the early detection of LR in rectal cancer after radical anterior resection with sigmoid-rectal anastomosis.

**Methods:**

We performed an anterior resection in 228 patients with stages I‑III rectal cancer who had undergone surgery from 2001 to 2008 in the Oncology Center in Bydgoszcz, Poland. Of these patients, 169 had perioperative radiotherapy or radiochemotherapy. All patients underwent PES with abdominal and pelvic imaging (abdominal ultrasound, computed tomography, magnetic resonance) and clinical examination. Sensitivities, specificities, positive likelihood ratios, negative likelihood ratios, and receiver operating characteristic curves were calculated to compare the value of colonoscopy versus imaging techniques for the diagnosis of LR.

**Results:**

During the 5-year follow-up, recurrences occurred in 49 (21%) patients; of these, 15 (6%) had LR, which was most often located outside the intestinal lumen (*n* = 10, 4%). Anastomotic LR occurred in 5 (2%) patients. The mean time to anastomotic LR was 30 months after initial surgery, similar to that of other locations (29 months). Both imaging and endoscopy were shown to be efficient techniques for the diagnosis of LR in anastomotic sites. In the study group, endoscopy did not provide any additional benefit in patients who were receiving radiation therapy.

**Conclusions:**

The benefit of PES for the detection of LR after curative treatment of rectal cancer is limited and not superior to imaging techniques. It remains a useful method, however, for the histopathological confirmation of suspected or confirmed recurrence.

**Supplementary Information:**

The online version contains supplementary material available at 10.1186/s12957-021-02413-0.

## Background

Colorectal cancer (CRC) is one of the most common malignancies worldwide. Its occurrence is associated with lifestyle, and, according to global data, it is expected to increase to an estimated 2.2 million new cases per year in 2030 [1]. Most patients with CRC will undergo radical treatment for the disease; they represent the third largest group of long-term cancer survivors [2]. At least 30% of CRCs are located in the rectum [3].

In 2017, 5617 Polish patients were diagnosed with cancer of the rectum and rectosigmoid junction [4]. Poland belongs to the group of countries with a medium risk of CRC, and the number of cases and cancer-related deaths is constantly increasing [1, 4].

**Fig. 1:**
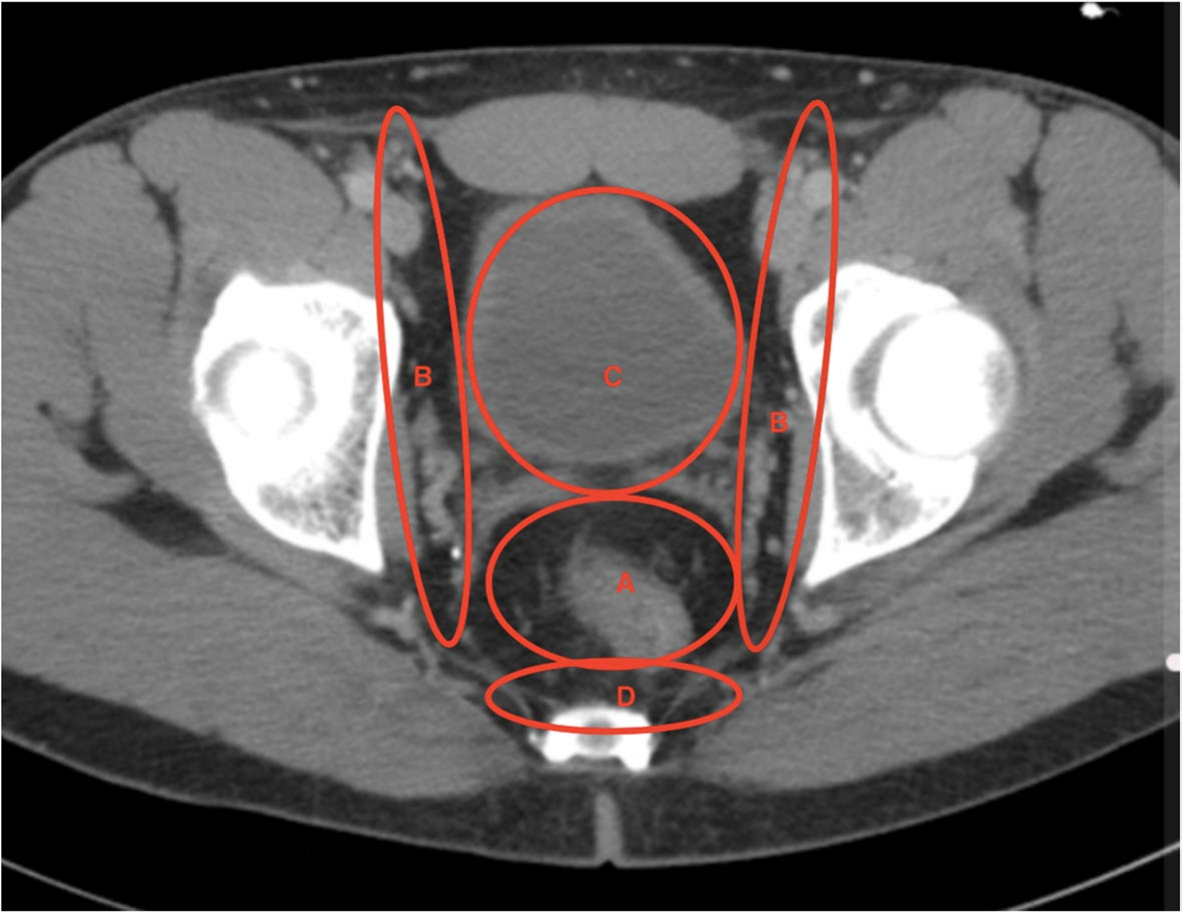
Anatomical localization of a local recurrence of rectal cancer, showing patterns of rectal cancer recurrence: (A) central (anastomotic site, perineal region, rest of mesorectum tissue), (B) lateral pelvic side wall, (C) anterior (genitourinary region, pubic bone), (D) posterior/presacral zone

Standard treatment of rectal cancer usually involves surgery, systemic therapies, and radiotherapy (RT) or chemoradiotherapy (CRT). The 5-year survival rates of patients undergoing radical therapy can reach 60% or more in developed countries regardless of stage at diagnosis; the prognosis varies significantly, however, depending on the initial stage of the disease [5–7]. The locoregional recurrence rate has decreased from about 30‑50% to 5‑10% as a result of precise qualification methods based on modern imaging, treatment that incorporates RT, and improved surgery with techniques such as total mesorectal excision (TME) [8–11]. Currently, most rectal cancer recurrence is systemic, not local. Nevertheless, local recurrence (LR) is still a major diagnostic and therapeutic problem in patients after radical treatment of rectal cancer, as LR significantly reduces the patient’s chances for long-lasting recovery. Diagnostic and therapeutic possibilities depend largely on the localization of the LR. Although there is no universally accepted classification of LRs according to their location, 4 typical LR zones are frequently distinguished: central/axial (anastomotic site, perianal region, rest of the mesorectum tissue), lateral (lateral pelvic sidewall: iliac vessels, lateral pelvic lymph nodes, sidewall musculature), anterior (genitourinary region, pubic bone), and posterior (presacral zone) (Fig. 1).

Although radical resection is commonly accepted as the best option to effectively treat LR of rectal cancer, this treatment is feasible (with a curative intent) in only a minority of patients [12, 13]. Oncological follow-up in patients after radical treatment aims to detect disease recurrence early and—theoretically—improve radical resection rates of non-advanced recurrence. However, the impact of intensive endoscopic and imaging surveillance on improvement of survival in patients with rectal cancer has not yet been unequivocally proven [14–16]. Moreover, the methods used for recurrence surveillance are still under discussion and currently not enough data are available to support their efficacy [17].

In this study, we aimed to clarify the clinical value of postoperative endoscopic surveillance (PES) for the early detection of LR in rectal cancer after radical surgery.

## Methods

### Patient selection

Between 2001 and 2008, 228 adult patients with pathological TNM (pTNM) stages I‑III of sporadic cancer of the rectum [18] underwent radical anterior resection with primary anastomosis in Oncology Center—Prof Franciszek Łukaszczyk Memorial Hospital, (Bydgoszcz, Poland). Patients were eligible for perioperative treatment according to the established principles described in Table 1. This retrospective study was approved by the Bioethical Committee at the Collegium Medicum Nicolaus Copernicus University.

**Table 1:** Framework for perioperative care of patients with rectal cancer (2001‑2008)

**Type of treatment**	**Resectability status**	**pTNM classification (MRI or CT)**	**Tumor localization**
Preoperative sRT^a^	Resectable	cT3 and/or N+	Middle or low rectum
Preoperative RT^b^ or preoperative CRT^c^	Unresectable or probably not R0	cT3-4 and/or N+	
No RT	Resectable, contraindications	cT1-2, N0	
		cT3 and/or N+	High rectum
Postoperative RT^d^ or postoperative CRT^e^	Not preoperative RT	pT3-4 and/or N+	
Postoperative CTx^f^		T4 and/or N+	

*Abbreviations*: *CRT* Chemoradiotherapy, *CT* Computed tomography, *CTx* Chemotherapy, *MRI* Magnetic resonance imaging, *pTNM* Pathological TNM classification, *RT* Radiotherapy, *sRT* Short-course radiotherapy

^a^Short-course radiotherapy (5 × 5 Gy) followed by immediate surgery (< 10 days from the first radiation fraction)

^b^45‑50 Gy in 28 fractions; a boost with a further 5.4 Gy

^c^45‑50 Gy in 28 fractions; a boost with a further 5.4 Gy combined with 2 cycles of 5-fluorouracil

^d^45‑54 Gy per fraction

^e^1.8‑2.0 Gy per fraction combined with 4‑6 cycles of 5-fluorouracil

^f^6 cycles of 5-fluorouracil

### Treatment and follow-up

Patients with a tumor of the lower and middle part of the rectum underwent TME, whereas a partial mesorectal excision was performed for those with more proximal tumors (upper third of the rectum). All surgeries were performed with open procedures by experienced teams (7 senior and 3 junior surgeons supervised by senior). A protective stoma was not performed as standard procedure; rather, it was created only if an anastomotic leak was suspected following anastomosis (2 ileostomy, 2 colostomy). The overall 30-day perioperative mortality rate was 1.3% (3 patients).

A total of 169 patients (74%) received perioperative RT, of whom 149 (65%) had preoperative RT or CRT. One-fourth of the total group did not receive irradiation because of various patient-related factors, including previous RT for the pelvic region and lack of consent for RT. Short-course RT (sRT; 5 × 5 Gy) was the most common treatment approach, followed by immediate surgery (< 10 days from the first RT fraction). Ninety-one patients (40%) had stage III (ypTNM) disease at presentation. The characteristics of the patients who underwent surgery are presented in Table 2. After treatment, all patients remained under surveillance according to the scheme described in Table 3.

**Table 2:** Patient characteristics (*n* = 228)

Gender, female/male	*n* (%)	105/123 (46/54)
Age	median (range)	61.3 (33‑90)
Distance from the anus, cm	median (range)	8.9 (3‑15)
Stage (pTNM)	*n* (%)	
I		48 (21)
II		85 (37)
III		91 (40)
pCR		4 (2)
Perioperative treatment	*n* (%)	
No		59 (26)
Preoperative RT		116 (51)
Preoperative CRT		33 (14)
Postoperative RT		20 (9)
Anastomotic leak requiring reoperation within 30 days of surgery	*n* (%)	16 (7)
Perioperative mortality 30 days after surgery	*n* (%)	3 (1)

*Abbreviations*: *pCR* Pathological complete response; others, see Table 1

**Table 3:** Surveillance protocol after radical anterior resection for rectal cancer (2001‑2008)

**Procedure**	**For years 1‑2**	**For years 3‑5**
Physical examination, including the rectal	Every 3‑4 months	Every 6 months
CEA	Every 3‑4 months	Every 6 months
Chest X-ray	Every 12 months	
Abdominal ultrasound or CT of the abdominal cavity	Every 4 months	Every 6 months
CT or MRI of the pelvis	Up to 1‑2 examinations during observation	
Gastrointestinal endoscopy (sigmoidoscopy, colonoscopy)	Every 12 months	

*Abbreviations*: *CEA* Carcinoembryonic antigen; others, see Table 1

We established a diagnosis of LR based on confirmation of at least one of the following major criteria: (a) histological confirmation, (b) clear bone destruction, and (c) positron emission tomography/computed tomography (PET/CT) indicating local recurrence, and at least one of the following minor criteria: (a) progressive tissue mass, (b) infiltration of adjacent organs, (c) subsequent growth of tumor markers, and (d) typical appearance of recurrence on endoscopic ultrasound, CT, or magnetic resonance imaging (MRI) [19].

In the case of recurrence, patients were restaged in order to develop an appropriate treatment plan.

### Statistical analysis

Statistical analysis was conducted by using the Statistica version 13.3 software package (TIBCO Software Inc., www.statistica.io). Qualitative and continuous variables are described with the usual descriptive statistics: numbers and percentages or medians with range (minimum-maximum) and moda with inerquartile range, respectively.

Sensitivities, specificities, positive likelihood ratios, negative likelihood ratios, and area under receiver operating characteristic curves were calculated to compare the diagnostic value of colonoscopy versus that of imaging techniques. All analyses assumed a non-parametric distribution of predictors and cut-off value equal 1 meaning presence of recurrence. The significance level in the analyses was *P* ≤ 0.05.

Post hoc sample size calculations for ROC analyses were performed using MedCalc® Statistical Software version 20 (MedCalc Software Ltd., Ostend, Belgium; https://www.medcalc.org; 2021), assuming alpha (significance) = 0.05, beta (1-power) = 0.2, calculated area under curve, and observed negative/positive ratio. Null hypothesis value equal 1 was considered as equivalence of the colonoscopy vs. imaging examinations. In all analyses, except local recurrences in RTH+ group, sample size was sufficient to reject the null hypothesis of equivalence of both examinations. Detailed results are presented in Supplementary data.

## Results

### The effectiveness of treatment

At 5-year follow-up, recurrences of any type were detected in 49 (21%) patients, 15 (6%) of whom had LR (Table 4). In the group of patients who had preoperative sRT, LR was detected in only 2 (2%) of them within 5 years of resection of rectal cancer. Distant metastases were confirmed in 41 (18%) patients, 8 of whom had distant metastases associated with LR. In total, LR affected 11 (6%) of the patients treated with RT.

**Table 4:** Patient outcomes by radiotherapy type at 5-year follow-up

**Treatment**		***n* (100%)**	**LR**	**Distant metastases**	**Total relapses**
			***n* (%)**		
Preoperative RT	5 × 5 Gy	116	2 (2)	20 (17)	21 (18)
	CRT or RT 50.4 Gy	33	6 (18)	10 (30)	13 (39)
	Total	**149**	**8 (5)**	**30 (20)**	**34 (23)**
Postoperative RT		20	3 (15)	3 (15)	4 (20)
No RT		59	4 (7)	8 (14)	12 (20)
Total		**228**	**15 (7)**	**41 (18)**	**49 (21)**

*Abbreviations*: *LR* Local recurrence; others, see Table 1

LRs were most frequently found outside the intestinal lumen (*n* = 10, 4%): in the presacral region (*n* = 5), in the lateral zones of the pelvis (*n* = 4), and in the anterior region (*n* = 1). In this group of patients (*n* = 10), 4 (2%) had isolated LR. Among the 228 patients, 5 (2%) had LR in the anastomotic site, 4 of these LRs (1.7%) being isolated.

### Detection of LR

**Fig. 2:**
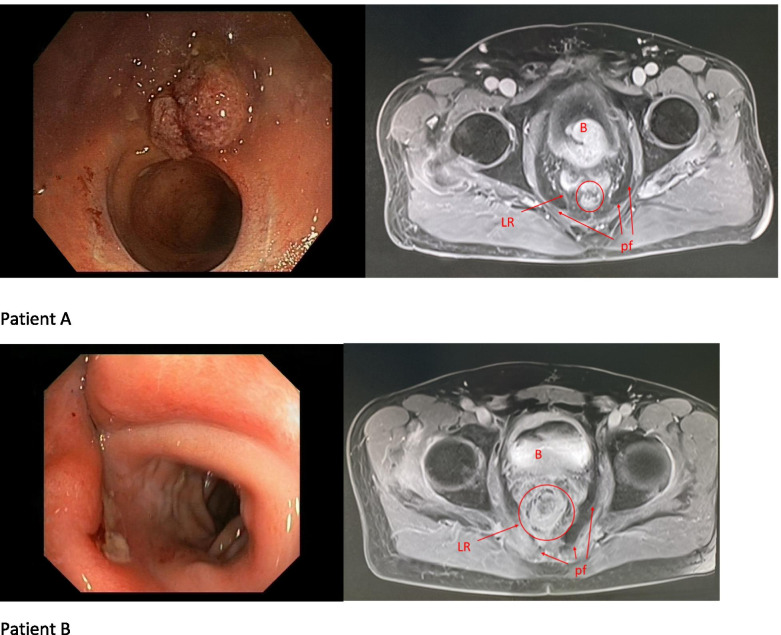
Intraluminal (patient A) and extraluminal LR (patient B). LR MRI shows LR in both patients, endoscopic examinations with histopathological verification only in patient A. Patient B after preoperative sRT. B, bladder; LR, local recurrence; pf, presacral fascia

In most cases, LR was not available for endoscopic examination (10 of 15; 67%). In these patients, the diagnosis of recurrence was made on the basis of imaging. After we analyzed the medical records, we found that endoscopic examination allowed for histopathological verification in 4 (of 5; 80%) patients with recurrence in the anastomosis. Only in 1 case was endoscopy the first examination to indicate the presence of LR; in the remaining 4 patients, endoscopy was performed after abnormal imaging results (imaging in these cases being the first indication of the presence of a recurrence) (Fig. 2). Time to diagnosis of recurrence from primary surgery did not differ between the intraluminal and non-intraluminal recurrence groups (30 months vs. 29 months).

The use of imaging techniques and endoscopy in the analyzed material was of similar effectiveness in diagnosing LR, although the results of these methods were not completely consistent. The specificity of colonoscopy was satisfactory (> 98% in all groups); however, its sensitivity was much lower than that of imaging (46.7% for all recurrences and 80% for anastomotic recurrences).

The effectiveness of imaging modalities in detecting recurrent rectal cancer did not differ significantly between groups of patients who did or did not undergo RT. Our analysis showed that colonoscopy was a good method for diagnosing recurrent rectal cancer in the anastomosis (area under the receiver operating characteristic curve > 0.8); however, it did not provide advantages over other diagnostic methods for diagnosing LR in patients who did not receive RT (*P* > 0.05 for both types of recurrences; Table 5).

**Table 5:** Results of ROC analysis assessing diagnostic power of colonoscopic vs. imaging techniques of investigation

	**All recurrences**						**Anastomotic recurrences**					
	AUC (95% CI)	Sensitivity	Specificity	*PPV*	*NPV*	*P* value	AUC (95% CI)	Sensitivity	Specificity	*PPV*	*NPV*	*P* value
Whole group	0.7310 (0.5644‑0.8795)	46.7%	99.5%	87.5%	96.4%	0.0066	0.8910 (0.6828‑1)	80.0%	98.2%	50.0%	99.5%	0.0002
RT+	0.7273 (0.5316‑0.9229)	45.5%	100.0%	100%	96.3%	0.0228	0.9910 (0.976‑1)	100.0%	98.2%	40.0%	100%	< 0.0001
RT-	0.7409 (0.4223‑1)	50.0%	98.2%	66.7%	96.4%	0.1383	0.8244 (0.496‑1)	66.7%	98.2%	66.7%	98.2%	0.0525

*Abbreviations*: *AUC* Area under the receiver operating characteristic (ROC) curve, *PPV* Positive predictive value, *NPV* Negative predictive value, *RT* Radiotherapy

## Discussion

Gastrointestinal endoscopy is used in the surveillance of patients after radical treatment of rectal cancer to identify and verify LR in order to increase the ultimate success rate. This method also enables clinicians to identify and remove metachronous tumors and precancerous lesions. Current guidelines recommend this examination as one of the foundations of surveillance. However, much of the evidence that forms the basis of these recommendations originates from outdated literature reported when patients were treated with various treatment regimens.

### Colon cancer, rectal cancer

The vast majority of published studies on postoperative surveillance have included patients with 2 separate entities: colon cancer and rectal cancer [15, 17, 20, 21]. Differences between these cancers include anatomical location (rectal cancer: retroperitoneal), diagnostic requirements (MRI, transrectal ultrasound), and therapies used (RT), which in turn may affect the diagnostic and therapeutic processes of the LR. Recurrent tumors located up to 8 cm from the sphincters are usually available by digital rectal examination and, above all, they show earlier clinical symptoms (altered bowel habits, hematochezia, abdominal pain).

### Risk of local recurrence

In most cases, colorectal LRs are localized outside the anastomosis [22–25], which may suggest a use for diagnosis imaging method, such as CT colonography [23]. Fuccio et al. [26] showed in the meta-analysis that the incidence of intraluminal LR in rectal cancer is 2 times higher than that in colon cancer. The authors reported that anastomotic LR virtually did not appear after 60‑72 months following surgical intervention.

Currently, less than 10% of patients who undergo radical treatment experience LR [27–29], owing to the use of an appropriate surgical technique (TME), the radical nature of the procedures (R0, circumferential resection margins: negative), and the combined treatments based on the RT schedule delivering a biologically effective dose above 30 Gy [30, 31]. Several studies have shown that about half of LRs are isolated, with no distant metastatic lesions [32, 33].

The risk of LR is associated with the following factors (among others): more advanced disease stage (American Joint Committee on Cancer/TNM), more distal location of the tumor, and perioperative treatment used. Preoperative RT reduces LR by approximately 50‑70% and postoperative RT by approximately 30‑40% in all locations of the rectum [34, 35]. This effect may be enhanced by the use of concurrent chemotherapy [36, 37], but are not observed, if adjuvant chemotherapy will be used after radiotherapy and radical resection [38, 39]. Some studies reported a significant reduction in the risk of LR in anastomosis after anterior rectal resection after the use of preoperative 5 × 5 Gy sRT [35].

### Synchronous and metachronous lesions

In patients with CRC, an estimated risk of the presence of synchronous neoplastic lesions is 2‑4% [40, 41]. Epidemiological data show that after radical treatment, patients with CRC have a 1.5- to 2-fold increased risk of developing metachronous lesions compared with that in a healthy population, as well as an increased risk (1‑2%) of developing a second primary CRC [42–44], especially in the first years after resection [41, 43]. The risk of developing metachronous adenoma after CRC resection can be estimated at less than 10% [45, 46], which is similar to that of developing adenomatous changes after polypectomy in the general population [47, 48].

### Postoperative surveillance

Improvement of overall survival in patients under postoperative surveillance after resection was confirmed in studies in which carcinoembryonic antigen testing, imaging (such as CT, MRI, PET/CT, or PET/MRI), and clinical visits were regularly performed in addition to endoscopic examination [49].

Imaging (CT, MRI) is a valuable and useful diagnostic tool for the diagnosis of LR. Ganeshan et al. [50] reported a sensitivity and specificity for CT of 76‑93% and 50‑100%, respectively; for fluorodeoxyglucose (FDG)-PET/CT of 94‑98% and 96‑98%, respectively; for MRI of 80‑91% and 86‑100%, respectively; and for FDG-PET/MRI of 94% and 94%, respectively.

The advantage of MRI over CT is that it allows better differentiation of the recurrent tumor tissue from fibrosis, of postoperative changes, and of changes after RT, with a sensitivity of 80–90% and a specificity of 100%, as has been described in the literature [51, 52] and also applies to other neoplasms [53].

Close monitoring of asymptomatic cancer patients allows for earlier detection of recurrence than does a diagnosis based solely on the presence of suspicious symptoms [52]. Nevertheless, the importance of extensive postoperative surveillance for recurrence after rectal cancer resection remains controversial. More recent publications indicate that intensified surveillance after surgery does not improve treatment outcomes [54–56]. PES remains only part of a multidisciplinary approach. A few studies that have investigated the effects of intensified follow-up endoscopy have consistently shown that, despite more frequent detection of asymptomatic recurrences and thus more frequent qualification for radical treatment, there was no improvement in overall survival in groups subjected to frequent endoscopic examinations [57].

### Guidelines

Earlier guidelines for post-rectal cancer surveillance included frequent endoscopic checkups of at least once every 6‑12 months [58, 59]. The currently recommended schemas, based on current publications on surveillance after radical treatment of rectal cancer, advocate examinations being done at least 2‑3 times over a 5-year follow-up period [60, 61], that is, much less often than previously recommended (Table 6). However, taking into account the clinical conditions that affect the likelihood of LR, such as the use of RT or the quality/radicality resection, it is possible to distinguish a group with a higher risk of intraluminal LR, which could allow individualized indications for intensifying PES. Identification of such groups is beyond the scope of our study and will require separate analysis of a larger amount of data, preferably coming from multicenter studies.

**Table 6:** Endoscopic surveillance described in current recommendations after radical surgery of rectal cancer with total mesorectal excision (visualized by endoscopic ultrasound or magnetic resonance imaging with contrast)

	CS I	CS II‑IV
ESMO Consensus Guidelines 2017 [60]	A completion colonoscopy within the first year if not done at the time of diagnostic workup (e.g., if obstruction was present) History and colonoscopy with resection of colonic polyps every 5 years up to the age of 75 years	
PTO/PTChO 2015 [59]	Colonoscopy at year 1 and then every 5 years; rectosigmoidoscopy every 6 months for 2‑5 years (in patients not undergoing radiotherapy or in the presence of T4 or N2 tumors)	
NCCN Consensus Guidelines 2021 [61]	Colonoscopy at the first year after surgery	Colonoscopy at year 1 after surgery; if no preoperative colonoscopy—in 3‑6 months after surgery
	If advanced adenoma—repeat in 1 year	
	If no advanced adenoma—repeat in 3 years, then every 5 years	

*Abbreviations*: *CS* Clinical stage, *ESMO* European Society for Medical Oncology, *NCCN* National Comprehensive Care Network, *PTO/PTChO* Polish Society of Oncology/Polish Society of Surgical Oncology

Our study has limitations because of its single-center and retrospective nature. However, the fact that patients were analyzed in one center contributes to the standardization of therapeutic and diagnostic procedures. The percentage of LRs in our analysis, including those located directly in the anastomosis, remained low (6.5%, 5 patients with anastomosis) and is similar to that reported by other studies [62–64]. The low recurrence rates are not conducive to reliable statistical analyses, although endoscopic examination is known to have a low sensitivity in detecting recurrences. Nonetheless, high specificity and the ability to sample biological material make endoscopy the preferred method for confirming the presence of recurrent lesions and verifying them histopathologically. Diagnosis of relapse is most often based on physical or imaging examinations (CT, MRI). Factors that increase the value of regular imaging tests as an alternative to endoscopy are the possibility of a simultaneous diagnosis of a lesion located outside the intestinal lumen and distant (systemic) lesions, as well as the diagnosis of possible consequences of radical treatment: postoperative fistulas, radiation-induced changes, and pelvic insufficiency fractures [65]. In addition, the invasiveness of endoscopic examinations should be taken into account, as they often result in poor patient tolerance associated with an increased risk of serious complications (including gastrointestinal perforation) [66]. Although small doses of radiation from X-rays that patients receive during imaging examinations (CT) have an impact on the body, the levels are too low to contraindicate even frequent examinations [56].

Our results do not confirm the advantage of PES in detecting recurrences in patients who are not receiving RT. This finding may have resulted from the small number of LRs detected (although a low rate of LR is the current standard). Given the results of other studies, however, a higher percentage of LRs and those located in the anastomosis can be suspected in this group of patients [34]. Although on the one hand, the use of RT reduces the number of LRs; on the other hand, it is recommended in more advanced tumors: in patients who are in general characterized as having a higher risk of LR, frequently located outside the bowel lumen. Thus, it remains debatable as to whether diagnostic indications for endoscopy in postoperative surveillance after rectal cancer treatment depend on the use of RT.

## Conclusions

Endoscopy of the gastrointestinal tract in patients under multidisciplinary surveillance after radical treatment for rectal cancer remains a useful diagnostic test that allows for histopathological confirmation of LR. However, because most recurrences are located outside the intestinal lumen and because of the higher sensitivity of imaging examinations such as CT or MRI, the role of endoscopy seems to be limited. Both our own results and the updated recommendations of oncological associations confirm this hypothesis, also taking into account the risk of the presence of metachronous lesions, which are better diagnosed with modern imaging techniques. We conclude that imaging studies in the follow-up of patients with rectal cancer should play a leading role, whereas endoscopy—although necessary—should be regarded as an additional and supplementary modality limited mainly to the intraluminal inspection and verification of imaging-diagnosed lesions.

## Supplementary Information

**Additional file 1: Figure 1s.** ROC analysis assessing diagnostic power of colonoscopic vs. imaging techniques of investigation in all recurrences and anastomotic recurrences groups. A, B: whole group. C, D: RT+ group. E, F: RT- group.

## Data Availability

All data generated or analyzed during this study are included in this published article.

## Abbreviations

CRC: Colorectal cancer
CRT: Chemoradiotherapy
CT: Computed tomography
LR: Local recurrence
MRI: Magnetic resonance imaging
PES: Postoperative endoscopic surveillance
PET: Positron emission tomography
pTNM: Pathological TNM classification
RT: Radiotherapy
sRT: Short-course radiotherapy
TME: Total mesorectal excision
