# Supplementary figures and images for: Efficacy of endoscopic surveillance in the detection of local recurrence after radical rectal cancer surgery is limited? A retrospective study
Source: World J Surg Oncol. 2021 Oct 21;19:308. doi: 10.1186/s12957-021-02413-0 (PMC8529797; doi:10.1186/s12957-021-02413-0)

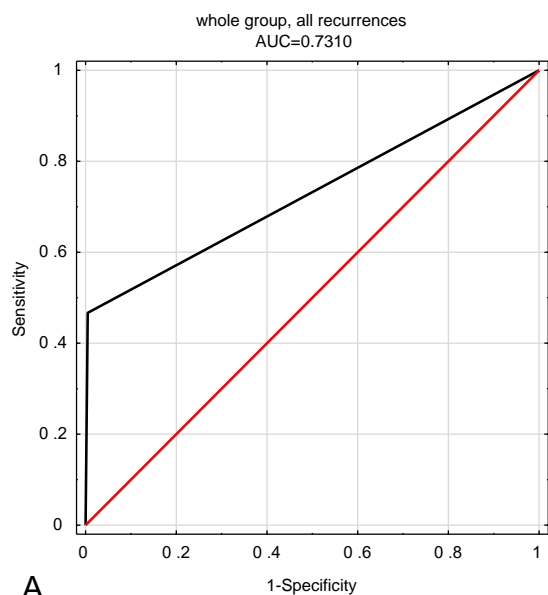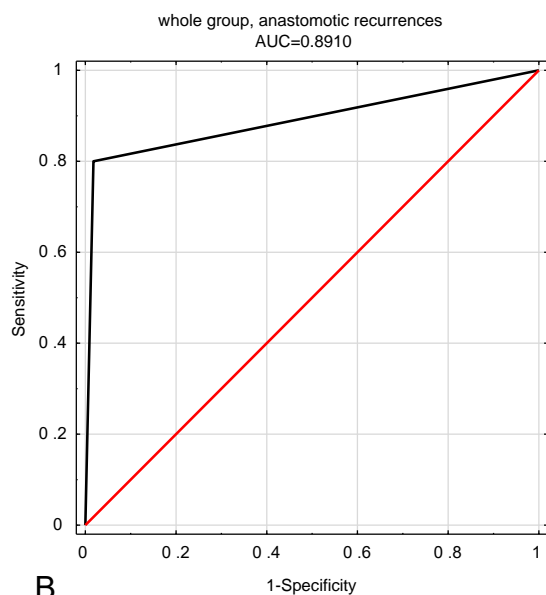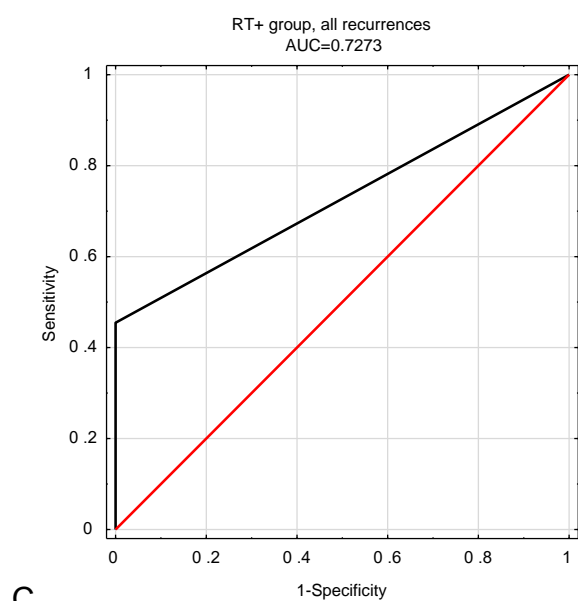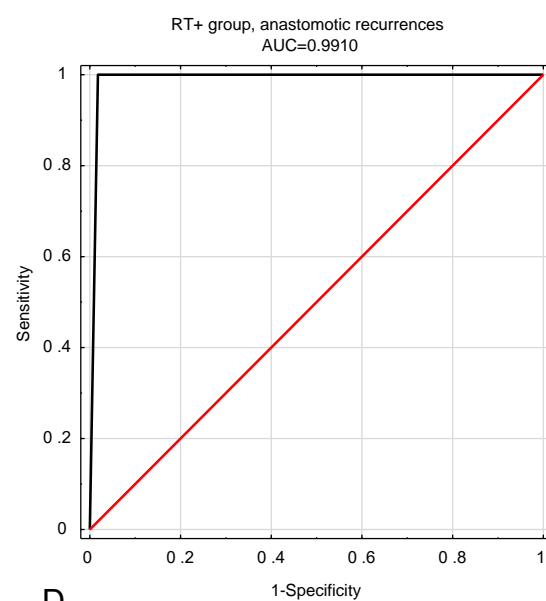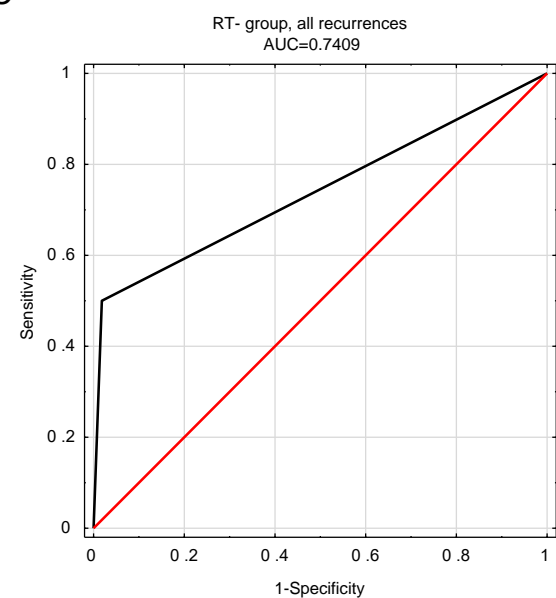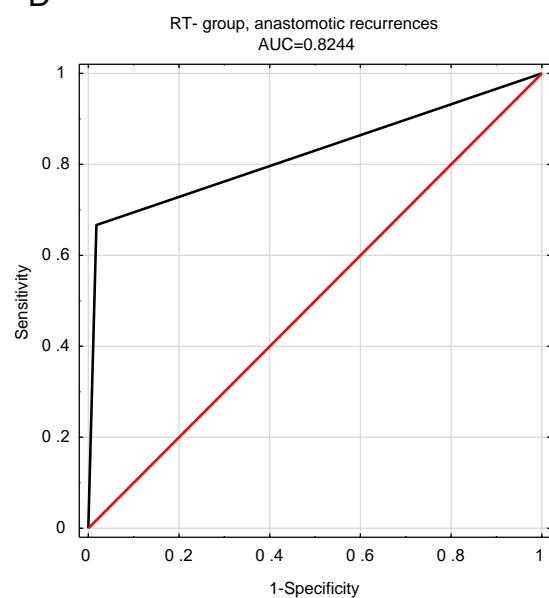

Supplement: Supplementary file 1 — Additional file 1: Figure 1s. ROC analysis assessing diagnostic power of colonoscopic vs. imaging techniques of investigation in all recurrences and anastomotic recurrences groups. A, B: whole group. C, D: RT+ group. E, F: RT- group. [file 12957_2021_2413_MOESM1_ESM.pdf]
